# Pasteur’s Experiments

**Published:** 1882-03

**Authors:** Rollin R. Gregg

**Affiliations:** Buffalo, N. Y.


### PASTEUR'S EXPERIMENTS.

### BACTERIA IN VARIOUS DISEASES.

ROLLIN R. GREGG, M. D., BUFFALO, N. Y.

The following paper was suggested by reading, in a recent number of a medical journal, a synopsis of Prof. Pasteur's paper upon "Vaccination in relation to Chicken Cholera and Splenic Fever," as delivered by him before the late International Medical Congress in London.

But before the reader can see and appreciate the full force and bearing of what is to follow, he must first know something of the essential facts in the Professor's experiments (if not already familiar with them), and his deductions therefrom, which are given in a condensed form below:

Prof. Pasteur first inoculates healthy chickens, each with the tiniest drop of the blood of another chicken, about to die of chicken cholera, to show the great virulence of that poison. The result is, of course, that the disease is communicated to the healthy fowl, and speedy death follows to every chicken so inoculated.

Next he prepares some broth from a healthy chicken, and fills a great number of small vases, or vials, with this broth, and numbers them, say from one to a hundred, or even to a thousand; then he dips the end of a very delicate glass rod into the blood of a chicken suffering from chicken cholera, and charges the broth in No. 1 vial with the minute drop of blood on the point of the glass rod. "In a short time," he says, "you will see the liquid," in that vial, "become turbid and full of tiny *microbes*." Now, "take from this vase," or from No. 1 vial, "a drop as small as you please, no more than can be carried on the point of a glass rod as sharp as a needle, and touch with this point a fresh quantity" of broth; that is, the broth in No. 2 vial, and precisely the same result follows to it as did to that in No. 1 vial, to which the drop of diseased blood was added—it also becoming "turbid and full of tiny *microbes.*" Then take as minute a drop from this second vial, and transfer it to a third vial of broth, and the same from the third to charge the fourth, from the fourth to the fifth, and so on, as high as you may go, the result is the same. Or, in the words of Prof. Pasteur: "You deal in the same way with a third culture-vase, with a fourth, and so on to a hundred or even to a thousand, and invariably, within a few hours, the culture-liquid becomes turbid and filled with the same minute organism."

And now we come to the most astounding part of all the results of his work, namely: That a healthy chicken inoculated with broth from the hundredth or thousandth vial so treated, will die as quickly and with the same symptoms as one inoculated with the fresh blood of the disease. Or, to give his exact language again, that there may be no question as to his assertions: "Let us take one of our series of preparations—the hundredth or the thousandth for instance—and compare it, in respect to its virulence, with the blood of a fowl which has died of cholera; in other words, let us inoculate under the skin of ten fowls, for instance, each with a tiny drop of infectious blood, and ten others with a similar quantity of the liquid in which the deposit has first been shaken up; strange to say, the last ten fowls will die as quickly, and with the same symptoms as the former ten; the blood of all will be found to contain, after death, the same minute infectious organisms." Upon the question as to what these alleged "minute infectious organisms" are, rests much of the real science of this whole subject, as we shall see further on, but it will be better, or more instructive, to clear up one or two other points before proceeding to that.

What has been quoted and said above relates wholly, as will be seen, to the inoculation and death to all fowls so dealt with, whether with the fresh and undiluted blood, or with the hundredth or thousandth successive dilution, or attenuation of that. Now comes up the question of vaccination and *prevention* of the disease, with broth treated, or charged in a similar manner with the same virus, with this difference and this only in manipulating it; namely, that he allows a fortnight or more to elapse between the charging of one vial and that of the next from that, and so on. By taking this course, or allowing such an interval of time between the charging of each of several successive vials, he modifies the deadly poison of this virus, and finally converts it into a great prophylactic, the vaccination of healthy fowls with which, will prevent them from taking the disease, in every case, as effectually as ordinary vaccine prevents small-pox.

He pursues a similar course with the virus of splenic fever, in successive vials of broth, which destroys every animal inoculated with it, in however high a dilution, the same as the blood of the disease itself, and as is the case in chicken cholera; unless he allows quite an interval of time to elapse between several successive dilutions, in which case it also is converted into a prophylactic against that fever, and so prevents the disease in every animal, whether horses, sheep, or cattle, vaccinated with it. And, in France alone, he claims that twenty million francs will be saved annually in the value of live stock by it.

Now, one point more and we are then ready for the summing up of the whole subject. Whether inoculating with the fresh diseased blood, or the deadly diluted virus propared from it; or whether vaccinating with that which has been manipulated into a prophylactic, from the latter, by the process described, one specific result follows in all cases, whatever else may come; and that is, the development of "microbes," in great numbers, in the part inoculated, or vaccinated, and in the animal's blood throughout the whole system, as well—though probably in much greater numbers, in case of inoculation with the deadly virus, than in vaccinating with the prophylactic, but this point is not clearly stated.

This brings us then to one of the most important of all questions, for a better and more scientific understanding of this subject, and that is: What are these microbes? Prof. Pasteur says they are living organisms, bacteria, or vegetable parasites, and all investigators and writers, not only upon these diseases, but on diphtheria as well, assert the same. But have not all such observers overlooked one *ever-present* and very important fact in all these and similar cases, and that the fact, that in every instance where blood congests, as the result of their inoculation, the *fibrin* in the blood of the *animal inoculated* commences at once, or soon, to coagulate, locally at first, and then more or less throughout the whole system, into minute granules as the result of the poison introduced; and that these minute granules of fibrin have been mistaken by them for living organisms, or vegetable parasites? Let us consider the following facts and see:

*Everything in nature begets its kind.* This is an axiom that none will deny. So constant and universal indeed is it, that many things which cannot beget their kind, properly speaking, nevertheless *induce* their kind. The least particle of the coagulum of sour milk, for instance, added to fresh, sweet milk, begins at once the work that soon leads to the coagulation of that. The particle of the coagulum introduced does not in and of itself grow, or multiply indefinitely, but simply induces the change in the corresponding element, caseine, of the fresh milk, that originally led to its own coagulation. Consider, also, in this connection, a few facts about pus-poisoning or pyæmia. A small quantity of pus, or of pus-corpuscles, taken up by or introduced into the blood and general circulation leads to the formation and deposit of pus in many parts of the system, that is, causes pyæmia; but the pus-corpuscles taken up do not beget the other corpuscles that follow, but they induce them; that is, set up a morbid action that leads to the decolorization of red blood-corpuscles, and their change into pus-corpuscles in a similar way to that by which other red corpuscles had been decolorized to make the pus-corpuscles first introduced. This general fact of things inducing their kind, might be enlarged upon almost *ad infinitum* in every department of nature, but let us here confine it to one specific result in disease.

The particles, molecular granules and fibrils, of coagulating fibrin (which have been mistakenly called bacteria), when added to a fluid like the broth, or the blood, that holds other fibrin in solution in it, induces a coagulation of the fibrin in that broth, or blood. The added particles of fibrin do not grow or multiply of themselves, in such enormous numbers as claimed for bacteria, nor at all. They simply induce the like change (coagulation) they had themselves previously undergone, in their like substance, the fibrin of the blood, or broth, to which they are added. This fact is here applied, it should be understood, either to the inoculation into the blood of healthy animals, some of the blood from diseased animals (or some vitiated broth), that contains coagulating particles of fibrin; or to the treating of the juices or broths from the flesh of healthy animals, by the diseased blood, or vitiated broth, after those juices have been manipulated by heat so as to prevent the fibrin in them from coagulating naturally.

That such are the *facts* in regard to coagulated particles of fibrin, inducing other fibrin, held in solution in any fluid, to also coagulate, is shown by the following from Carpenter's Physiology, page 60, where, in speaking upon the fibrillation of fibrin, he says:

"This *fibrillation* seems to bear a certain analogy to crystallization, being the result of forces which tend to withdraw the solid particles from their state of solution in the liquid, and to bring them together in a certain definite mode of aggregation; and there are certain peculiarities in the process which to some extent bear out this analogy. Thus, just as crystals will form around a nucleus of the same kind, from a solution which *would not otherwise have deposited them*, so will a fibrinous coagulum often separate from a serous fluid, and form around a piece of washed clot of blood, or of the buffy coat, or of muscle or some other animal tissue placed in it, although the fluid (such as that of hydrocele) *would not have othewwise shown any disposition to coagulate.*" (The italics are our own to show more distinctly the facts in this respect as they stand.)

The remarkable persistency in the coagulating power of fibrin, whenever, and so long as any of it remains in solution in any fluid, may be seen from the following from Virchow's Cellular Pathology, page 192:

"In many forms of pleurisy the exudation long remains fluid, and a number of years ago a peculiar case came under my notice, in which, on puncturing the thorax a liquid was evacuated which was perfectly clear and fluid, but in a short time after its evacuation had its whole mass pervaded by a coagulum, as is often the case with fluids from the abdominal cavity. After I had removed this coagulum from the liquid by stirring it, in order to convince myself of its identity with ordinary fibrin, the next day a fresh coagulum displayed itself, and this took place also on the following days. This coagulative power lasted fourteen days, although the operation had been performed in the midst of the heat of summer."

This shows what a remarkable thing coagulating fibrin is, and especially in connection with disease or diseased fluids.

Again, it should be borne in mind, that the molecular granules, the fibrils and spirals of coagulating fibrin, are, in their very appearance, and under all circumstances, precisely like the three classified forms* of bacteria, spherical, rod-like and spiral (the microscope

* From carefully reading numerous authors upon bacteria, I am confident that many, if not all of them, have also included in their lists of bacteria (but outside of the three "classified forms," named above, which are the three stages of coagulating fibrin) colored blood-corpuscles that have been *decolorized* by disease, but more especially the numerous granules that such decolorized corpuscles are constantly breaking up into, or bursting and thereby releasing, in connection with every disease where "bacteria" are found.

has never pointed out the slightest distinction between them), and that they occupy the same positions and demean themselves in precisely the same manner wherever found.

Therefore, if Prof. Pasteur will repeat his highly important experiments, recently reported in London, and while doing so, keep well in view the foregoing facts, he will no doubt be led to revise his conclusions, from seeing that his microbes, or bacteria, of chicken cholera, and of splenic fever, are simply coagulated particles of fibrin in the blood of the diseased animals, and that those caused in healthy animals by their inoculation with such blood, are also nothing but coagulating particles of the fibrin of their blood—the coagulation thereof being simply induced in the healthy animal by like matter, coagulated fibrin, in the diseaeed animal's blood, introduced by the inoculation.

The power of diseased or dead animal matter, to compel the coagulation of fibrin, even in the living body, may be seen by the following from Carpenter, page 197:

> "Again, the contact of dead animal matter with the blood, appears to promote the coagulation of its fibrin in a very remarkable degree; occasioning *coagula to form* whilst it is yet *actively moving* in the *vessels of the living body.* Thus, M. Dupuy found that the injection of cerebral* substance into the veins of an animal occasioned its death almost as instantaneously as if prussic acid had been administered; the circulation being rapidly brought to a stand by the formation of voluminous clots in the heart and large vessels. These experiments were repeated and confirmed by M. de Blainville. The same effect is produced with *still more potency* when the substance injected is rather *undergoing degradation* than actually dead." (The italics here also our own.)

If, then, a little healthy brain substance injected into the veins, will kill as quickly as a dose of prussic acid, by forming "voluminous clots" of fibrin in the blood; and if any animal matter, "undergoing degradation," though not yet dead, is still more powerful in this respect, we can better see and understand the power which the blood of a diseased animal (that already contains coagulating fibrin), must have in coagulating the fibrin in the blood of a healthy animal, inoculated with it.

In speaking of inflammation, the exudation and organization of fibrin in connection therewith, Wood, vol. I, page 28, says:

---

* "There is no reason to suppose that cerebral substance possesses a more special influence than would be exerted by any other tissue which could be as easily mixed up with the circulating current."—NOTE, BY CARPENTER.

"As it first escapes it is a homogeneous, formless, transparent fluid; but very soon afterwards, if examined by the microscope, it is found to contain *multitudes of fibrils*," and "*great numbers of minute granules* of different sizes." (Italics ours.)

Lehman, vol. I, page 312, speaking of the coagulation of fibrin under various circumstances, says:

"The same process goes on *within the vessels of the living organism, as soon as the blood ceases to circulate*." (Italics ours.)

And under *all* circumstances, when fibrin coagulates, it organizes first into "*great* numbers of granules," and these join together to form "multitudes of fibrils."

Therefore, of one thing all may be assured, and that is, in the inflammation excited in any part by inoculation with any, or especially with poisonous or diseased matter, there are always found in that part great, if not almost innumerable, numbers of very minute coagulated or molecular granules of fibrin; and it therefore becomes the duty of Prof. Pasteur to point out the clearest and most unmistakable distinction between these and his so-called bacteria, which are claimed to occupy the same parts, and something of the proportions of each, in the part, or we are fully warranted in assuming that all the latter are simply nothing more than particles of organized fibrin, coagulated there by the poison, or by the inflammation, as the case may be. He assumes and asserts the presence of an *unnatural* and *foreign* element, *vegetable* organism, in the blood, etc., without clear proof that they are such, while we can positively assert and *prove* the actual presence of a *normal* element, fibrin, there, but morbidly changed, that is, coagulated into minute particles, by the inoculating poison, or by the inflammation which that excites. Therefore, I repeat, the burden of proof lies wholly with him to make good his unnatural claim, or the natural *fact* must and should take its place.

Fibrin is in excess in the blood in cholera and in splenic fever, as it is in diphtheria, and as soon as that excess becomes at all pronounced, it commences to coagulate into molecular granules and fibrils in the blood of those diseases, just as it does in diphtheria; hence, when ever so little of that blood is introduced into healthy blood, it induces more or less of the like change in that, as already sufficiently shown; and *not* the growth of vegetable parasites, as claimed, in realms where vegetable life does not exist and could not live.

And all this enforces another important lesson upon the fact already claimed, namely, that everything must produce its kind. Inoculation with the blood, or the microbes, or the bacteria, that is, with the coagulating particles of fibrin of chicken cholera, will *not* produce splenic fever or diphtheria, nor will the inoculation with the alleged bacteria of splenic fever produce either diphtheria or chicken cholera, nor those of diphtheria either of the other diseases, but each its exact kind and that only.

And, per force, vaccination with the microbic grafts, that is, with the manipulated, or many-times-re-induced particles of coagulated fibrin, from either of these diseases, cannot prevent one of the others, any more than can the manipulated virus of small-pox prevent measles, or *vice versa;* which throws us back on the same fact, still again, that each virus produces, or is a prophylactic against, as the case may be, its own exact kind, and nothing else; and leads to the only other rational conclusion, namely, that these particles of fibrin carry with them the contagious principle of the disease into the blood of the healthy animal, as a separate and immaterial, or dynamic force, or poison, clinging to, or in some other way associated with them, and which is the cause of all the other results.

On the contrary, if all these germs, microbes, or bacteria, so-called, were vegetable parasites as claimed, and so exactly alike* as asserted, they should all produce the same or very similar results, or diseases —those of chicken cholera causing indiscriminately that, or diphtheria, or splenic fever, or either of these latter chicken cholera, which is *never* in the slightest degree the case. Or, rather, were they all vegetable and exactly alike, or belonging to the same closely related family, as they must, if they were that, it is impossible to see how there could be any difference that would be discernible in their effects. But being coagulated particles of fibrin and herein exactly alike, they may and do induce their exact kind in causing other fibrin to coagulate, but there the similarity stops, and still leaves the specific dynamic force, or poison of each disease, to be accounted for, which, as already said, they in some way carry along with them,

* "I will not trouble you with any attempt to describe the differences which these organisms present, for subsequent experience has shown that the *most deadly* may be *undistinguishable* from the *most harmless*, by any *detectable physical peculiarity.*" (Italics ours).—Extract from address by Charles Cameron, M. D., LL. D., M. P., President of the Section, delivered in the Health Department of the Social Science Congress.

and which every time produces its own specific results and those only, whether of diphtheria, cholera, splenic fever or other contagious disease.

Organized fibrin, too, is one of the most indestructible of all the soft tissues, nothing but bone exceeding its qualities in this respect. Thus, "Mr. Hatchett macerated three pounds of muscle, cut into thin pieces, for fifteen days in cold water, changed every day, pressure being also applied; he afterwards *boiled* it for *five hours every day* for *three weeks*, changing the water as before; and finally it was pressed and dried by the heat of a water bath;" and all this in order to get a pure specimen of fibrin. Hence, is it surprising that Prof. Pasteur found, as he says, his deadly "microbes," (coagulated particles of fibrin), in pits where animals dead from splenic fever had been buried several years? And is there anything *vegetable*, in so minute a form, that would last so long buried in the ground, and under the heat, moisture and frosts of the changing seasons?

The presentation of this subject here, or in this manner, must not be understood as being done to obtrude mere individual opinions, or as captious criticism, as it is neither. It is simply presenting a connected series of *scientific* facts, of which there are many more, which should and *must* have a hearing before we can settle upon the true nature of diphtheria, or of the other diseases named.

Is it not a little suggestive that Prof. Pasteur's microbes require, the same action of oxygen, or exposure to the air, to develop them, that is required by fibrin to coagulate it, as seen in drawn blood and in dropsical fluids, containing fibrin?

"Without oxygen bacteria cannot live," says Prof. Bollinger, another high authority. Without oxygen fibrin cannot coagulate,* I respond, as we see in dropsical effusions into the chest, abdomen, etc., where neither air nor even the oxygen of the blood can reach them, and they do not coagulate until exposed to the air, then they do.

"The whole development of these organisms, and especially the

* Even in those cases, cited by Carpenter, where other materials than oxygen were used to coagulate the fibrin, there was exposure to the air also as a part of the process, excepting when it was coagulated in the vessels of the living body, and here certainly there was the oxygen in the blood to aid in the work, as there is in all cases of inoculation; so it may be that oxygen is always one of the requisites to such coagulation, though whether this be true or not, it does not destroy, or even disturb any of the other facts."

formation of spores, is completed on the surface of the fluids, and under the influence of an abundant supply of oxygen," says Prof. Klebs, of speaking of baccilla, or rod-like bacteria. Fibrin comm nces its coagulation on the surface of fluids that contain it, I answer; and so look where we may the comparisons and similarities are complete.

---

CUTANEOUS ERUPTIONS CAUSED BY THE USE OF CERTAIN MEDICINES (*Giorn. It. dei Malatt. Vener. e del Pelle*, June 1881). Anspitz, in his valuable "System der Hautkrankheiten," gives the following list of eruptions liable to follow the use of certain remedies. It will be a useful table for reference:

*Quinine.*—(*a*) Scarlatinous erythema, (*b*) morbillous papular erythema, (*c*) hæmorrhagia and purpura, (*d*) wheals, œdema, pruritus.

*Cinchona. Belladonna, Strychnine, and Stramonium.*—Manifestations like papulæ sudorales.

*Digitalis.*—Erythema after a few days' use.

*Aconite.*—Vesicular exanthema.

*Santonine.*—Vesicles, wheals.

*Rhus Venenata and Toxicodendron.*—Vesicular eruption.

*Opium and Morphine.*—Erythema, papular eruption, with much desquamation and pruritus.

*Pilocarpin* (?)—Augmentation of the perspiration.

*Phosphorus.*—Purpura.

*Phosphoric Acid.*—Bullous eruption.

*Mercury* (internally).—Erythema, eczema.

*Arsenic.*—Erythema and papules, eczema.

*Carbolic Acid.*—Erythema, vesicles, or wheals.

*Salicylic Acid.*—Purpura, vesicles with laryngeal catarrh, wheals.

*Chloral Hydrate.*—Erythema (well colored), pruritus, desquamation, purpura and petechiæ, eczema with crust and scab.

*Balsam Copaiba, Cubebs, Turpentine.*—Vesicles, erythema, eczema.

*Cod-Liver Oil.*—Acne.

*Iodide of Potash.*—Papules, vesicles and bullæ, pustules and ecthyma, eczema, ecchymosis and purpura.

*Bromide of Potassium.*—Papules and pustules, deep tubercles and ecchymosis, vesicles, ulcers.—*Virginia Medical Monthly.*

